# Impacts of Extraction Methods in the Rapid Determination of Atrazine Residues in Foods using Supercritical Fluid Chromatography and Enzyme-Linked Immunosorbent Assay: Microwave Solvent vs. Supercritical Fluid Extractions

**DOI:** 10.1100/tsw.2005.7

**Published:** 2005-01-14

**Authors:** Mohamed H. El-Saeid, Ijeoma Kanu, Ebere C. Anyanwu, Mahmoud A. Saleh

**Affiliations:** ^1^ Department of Chemistry and Environmental Toxicology, 3100 Cleburne Avenue, Texas Southern University, Houston, Texas, USA; ^2^ Department of Microbiology, Abia State University, Uturu, Nigeria

**Keywords:** toxicology, pesticides, imported fruits and vegetables, SFE and SFC method

## Abstract

It is an accepted fact that many food products that we eat today have the possibility of being contaminated by various chemicals used from planting to processing. These chemicals have been shown to cause illnesses for which some concerned government agencies have instituted regulatory mechanisms to minimize the risks and the effects on humans. It is for these concerns that reliable and accurate rapid determination techniques are needed to effect proper regulatory standards for the protection of people's nutritional health. This paper, therefore, reports the comparative evaluation of the extraction methods in the determination of atrazine (commonly used in agricultural as a herbicide) residues in foods using supercritical fluid chromatography (SFC) and enzyme-linked immunosorbent assay (ELISA) techniques. Supercritical fluid extraction (SFE) and microwave solvent extraction (MSE) methods were used to test samples of frozen vegetables, fruit juice, and jam from local food markets in Houston. Results showed a high recovery percentage of atrazine residues using supercritical fluid coupled with ELISA and SFC than with MSE. Comparatively, however, atrazine was detected 90.9 and 54.5% using SFC and ELISA techniques, respectively. ELISA technique was, however, less time consuming, lower in cost, and more sensitive with low detection limit of atrazine residues than SFC technique.

## INTRODUCTION

Atrazine is a selective pre- and early postemergence herbicide. It has been found in surface and ground water as a result of its mobility in soil. It is relatively stable in soil and aquatic environments, with a half-life measured in months, but degraded by photolysis and microbial degradation in soil. The way that many farmers are using atrazine in their cropping program creates avenues for food contamination and eventual health risks. Hence, this farming process is being reviewed and could lead to changes in the way these chemicals are used in the future. In addition, atrazine residues are now regularly being detected in numerous water bore and surface water catchments in country towns [1,2]. This means that atrazine has the potential to contaminate groundwater because of its slight water solubility, long half-life, and sorption to organic matter.

Hence, enzyme-linked immunosorbent assay (ELISA) has gained acceptance as a technique for the rapid screening as it is quantitative, easy to use, allows many samples to be run, and is less expensive for the determination of atrazine herbicide residues[3,4,5]. The U.S. Department of Agriculture (USDA), U.S. Environmental Protection Agency (USEPA), and U.S. Food and Drug Administration (USFDA) investigated and approved the application of immunoassays for the detection of residues in foods based on its innovative technological method, method reproducibility, sensitivity, function of the affinity and selectivity of an antibody for an antigen can be measured, which ultimately yield good results when compared with other traditional analyses [6,7]. Most recent publications describe the determination of pesticides including atrazine in food matrices demonstrating that supercritical fluid extraction (SFE) is a viable extraction technique that is less time consuming, less solvent, and has higher cost savings over the conventional extraction when coupled with ELISA techniques[7,8,9,10,11]. In this investigation, four such techniques — SFE and microwave solvent extraction (MSE) for quick extraction; supercritical fluid chromatography (SFC) and ELISA for quick determination of atrazine residues in row and spiked food matrices — are comparatively used. Frozen vegetables, fruit juice, and jam samples from local food markets in Houston were analyzed for the atrazine herbicide residue contents.

## METHODS

Using SFE, MSE, SFC, and ELISA, food samples from eleven different types of fruit juice (guava, mango, and fruit cocktail), frozen vegetable (mixed, molukhia, artichoke, colocassia, aubergines, beans, and okra), and strawberry jam samples were obtained from Houston local food markets. The preparation of each food sample was first homogenized, then divided into two portions, one as a blank or control sample for quality control and quality assurance, and the other for spiking atrazine herbicide. Both of these samples were subjected to freeze drying. The frozen, dried, powdered food samples then were stored in 1-kg glass containers at –20ºC until analysis. To calculate relative standard deviation (RSD%), four replicate spikes were analyzed.

### Recovery Assays

Ten untreated food samples were spiked with atrazine. Recovery assays were performed at levels ranging from 0.1–5.0 µg/g. The spiked samples were then homogenized and allowed to equilibrate for 1 h prior to freeze drying. Four replicates were analyzed to calculate the recovery and RSD% by both extraction and determination methods.

## Atrazine Kit and Standard

EnviroGard Triazine Plate Kit 72110 was obtained from Strategic Diagnostics Inc. (Newark, DE). Triazine Plate Kit contains 8 strips of 12 antibody-coated wells each in a strip holder; one vile of each of the following items: negative control (0.0 ppb atrazine), atrazine-enzyme conjugate, substance, chromogen, and stop solution; and vile of each 0.01, 0.25, and 2.0 ppb atrazine calibrator. Triazine Plate Kit was stored at 4–8ºC until analysis. Atrazine standard commonly used for the agricultural purposes as an herbicide was obtained from Chemservice, Inc. (West Chester, PA).

## Extraction 1

### *SFE*

The atrazine extraction method[13,14,15,16] was modified and used in the first part of the investigation. This involved the use of the SFE equipment model 7680 T (Hewlett-Packard), which included an automated available restrictor and a solid phase sorbing trap prepackaged with 30 Fm of Hypersil ODS into which the $CO_2$ extraction solvent was decompressed during collection. Five grams of dry food samples were transferred into the extraction thimble. The extraction process was carried out in three steps. In the first step, $CO_2$ density was 0.25 g/ml, at an extraction pressure of 77 bar (1117 psi), chamber temperature 40EC, and $CO_2$ flow rate of 1.0 ml/min. In the second step, $CO_2$ density was 0.67 g/ml, at an extraction pressure of 239 bar (3469 psi), chamber temperature 80EC, and $CO_2$ flow rate of 2.5 ml/min. The nozzle temperature was 45EC in both steps. The sample extract was collected on the ODS sorbing trap at 10EC. The extracted sample was eluted from the trap with 1.5 ml of methanol at flow rate of 0.4 ml/min and a trap temperature of 40EC and collected in auto sampler vials placed in a fraction collector.

The ODS trap was regenerated between extractions by rinsing with 2 ml of methylene chloride followed by 2 ml of methanol at 1 ml/min. The same conditions were applied in the third step as to the second step except that a 30% modifier was used. The time of extraction per sample was 55 min (step one 5 min, step two 40 min, and step three 10 min). The first step was performed to eliminate hydrocarbons and nonpolar compounds, the second step was performed to extract the insecticides, and the third step was performed to wash the thimble and ODS trap and to insure that no atrazine residues escaped in step 2.

### *MSE*

The USEPA application note No. E009 was modified[16,17,18,19,20] and used in this study. MSE system model MES-1000 with lined extraction vessels (LEV)[14,15,16,17,18,19,20] was involved. This system consists of a 950-W microwave instrument, which has been specifically designed for use with organic solvents. Extraction conditions are controlled by temperature using an inboard fiber optic system, which allows extraction temperatures to be selected from 20–200ºC in 1ºC increments. Temperature and pressure data can be copied to an external printer or downloaded to a PC; a maximum of 12 samples can be extracted simultaneously. Safety features of this system are intended to prevent ignition of flammable and explosive extraction solvents, all ignition sources have been eliminated from the microwave cavity, the cavity is Teflon lined, and additional Teflon has been added to the cavity ceiling. Other safety features include a solvent vapor detector in the system air exhaust, which turns off the microwave magnetron if solvent vapors are detected in the microwave cavity. An exhaust blower continually moves air through the cavity. All extraction vessels are connected to a sealed center collection vessel so that in the event of vessel safety membrane rupture, solvent vapors will be contained, directed from the cavity through a venture exhaust tube, and routed to the external exhaust. The venture in the cavity exhaust creates a slight vacuum that helps to remove solvent vapors. The extraction vessel is a double-walled vessel specifically adapted for use with organic solvents. The vessel consists of an inner Teflon PFA liner and liner cover that contain the 5-g food sample and 50 ml (Acetone-Hexane 3:2) extraction solvent, and the outer % Bound for the zero (BO). that gives mechanical strength to the vessel is a special grade of Ultem polyetherimide that is resistant to attack by organic solvents. The vessel has a working range of 120ºC, 80 psi; microwave power was 70% and time of extraction 30 min.

## Analytical Techniques

### *SFC*

In these analytic techniques, the atrazine herbicide determination methods[18] were also modified[13,16] to suit the requirement of the present investigation. A Hewlett-Packard SFC model G 1205A attached to an HP 1050 diode array detector, modifier pump G 1205A, and a silica column (Alltec Hypersil APS 25 µm, length 205 mm, ID 4.6 mm) was used. Atrazine herbicide was run at an oven temperature of 30EC, pressure 80–150 bar (30 bar/min), flow rate 1–2 ml/min, modifier 2–3% methanol (5%/min) and detected at 220 nm. These methods were elaborated from trials of several injections to obtain the best and fast separation of atrazine.

### *ELISA*

The EnviroGard Triazine Plate Kit was used in this technique. It is a quantitative laboratory test for the detection of atrazine residues in water and foods and it uses polyclonal antibodies, which bind atrazine-enzyme conjugates for a limited number of antibody binding sites. Antibodies that bind atrazine compound are immobilized to the inside of the wells.

## Assay Procedures

The assay procedure steps were as follow:

1. Plan the strip format allowing for the placement of negative control (C), three calibrators (C1–C3), and the other for food samples extract (S1–S88).
2. Add 80 µl of negative control (C) using multichannel pipette, 80 µl of each calibrator (C1–C3), and 80 µl of each food samples extract (S1–S88) to their respective wells.
3. Add two drops (80 µl) of atrazine-enzyme conjugate to each well.
4. Move the strip holder in the rapid circular motion on the benchtop for 1 min to mix the contents of wells thoroughly.
5. Cover the wells with parafilm to prevent evaporation and incubate at ambient temperature for 1 h with orbital mixing at 200 rpm.
6. Remove the covering and vigorously shake the contents of the wells into a sink, five times washing by a microtiter plate washer to flood the wells completely with cool running tap water, then shake to empty.
7. Add two drops (80 µl) of substrate to each well beginning with the negative control (C) and calibrators (C1–C3) and finishing with the food samples extract wells (S1–S88).
8. Add two drops (40 µl) of chromogen solution to each well in the same order as for substrate.
9. Mix the contents of the wells by the rapid circular motion on the benchtop for 1 min and cover the wells by parafilm to prevent evaporation and incubate at ambient temperature for 30 min with orbital mixing at 200 rpm.

10. Add one drop (40 µl) of stop solution to each well, arrest the blue color development, and turn the reaction solution yellow, mix thoroughly without spilling until all of the blue has converted to yellow.

### Spectrophotometric Measurements

A microtiter plate reader system model MR-5000 with Biolinx assay management software (Dynatech Lbaboratories Inc, Matthews, Chantilly, VA) is used as microtiter plate reader to interpret the results at wavelength 450 nm auto-zero on air in a blank well to measure and record the optical density (OD) of each wells contents as % Bound for the zero (BO) using semi-log curve with data reduction capabilities fit for the standard curve.

### Calculations

After OD reading by microtiter plate reader for all of the 96 wells, the OD of each set of calibrators and food samples is averaged and used to calculate the Bo. The %Bo calculation is used as a means of equalizing different runs of assay, also %Bo relationship of calibrators and samples to the negative control were kept fairly constant. Graph of %Bo of each calibrator against its atrazine concentration on the semi-log scale was obtained. Then, the atrazine concentration of each food sample was obtained by finding the %Bo value and the corresponding concentration level on the graph. Interpolation of sample concentration is only valid if the %Bo of the sample falls within the range of the %Bo's set by the calibrators. Thus:

$$\%Bo = \text{Average OD of calibrator or sample/Average OD of negative control} \times 100$$

## RESULTS

Atrazine herbicide residues and RSD in food samples extracted by SFE and MSE, determined by SFC at 220 nm and ELISA technique at 450 nm in each freeze-dried food sample are presented in Table 1. A comparison of the two extraction methods showed that although both methods were effective, the ELISA was more effective than the SFE. The atrazine recovery and RSD percentages of spiked food samples extracted by SFE and MSE, determined by SFC at 220 nm and ELISA at 450 nm are shown in Table 2.

Applying SFE in multiresidues, which we thus developed to obtain the optimum conditions, it was possible to extract 35 pesticides in each freeze-dried food sample (control or spiked) in 55 min. The process was fast and there was no cleanup required. The detection limit (0.001 ppb) was also low when compared with SFC/Ultra Violet Detector (Table 3). It was observed that the minimum detection limits (MDL) of atrazine by SFC/UVD at 220 nm and ELISA technique at 450 nm were observed at all the concentrations (0.001–1.0) with ELISA, however, with SFC only 0.001 was not detected.

Comparing the overall extraction and determination time by SFE, MSE, SFC, and ELISA techniques on each food sample, we found 45.0, 2.7, 5, and 1, respectively (Table 4).

## DISCUSSION

The comparative evaluation of the extraction methods in the determination of atrazine residues in foods using SFC and ELISA techniques has been investigated. Our modified method gave a high recovery percentage of atrazine residues found using both extraction methods (90.9 and 54.5%, respectively). ELISA technique was, however, less time consuming, lower in cost, and more sensitive with low detection limit of atrazine residues than SFC technique.

**TABLE 1**
**Atrazine Residues (PPb) and RSD in Food Samples Extracted by SFE and MSE, Determined by SFC at 220 nm and ELISA Technique at 450 nm**

| Food Samples | SFE | | MSE | |
|---|---|---|---|---|
| | SFC | ELISA | SFC | ELISA |
| Guava juice | ND | 0.02 ± 03 | ND | 0.02 ± 03 |
| Mango juice | 0.31 ± 05 | 0.35 ± 08 | 0.27 ± 07 | 0.34 ± 05 |
| Fruit cocktail juice | ND | ND | ND | ND |
| Frozen mixed vegetable | 0.72 ± 08 | 0.76 ± 08 | 0.70 ± 09 | 0.74 ± 08 |
| Frozen molukhia | 0.61 ± 04 | 0.69 ± 03 | 0.58 ± 03 | 0.67 ± 02 |
| Frozen artichoke | ND | 0.02 ± 08 | ND | 0.02 ± 08 |
| Frozen colocassia | ND | 0.03 ± 03 | ND | 0.02 ± 03 |
| Frozen beans | 0.61 ± 06 | 0.71 ± 08 | 0.59 ± 08 | 0.64 ± 08 |
| Frozen aubergines | 0.24 ± 04 | 0.32 ± 03 | 0.22 ± 07 | 0.29 ± 03 |
| Frozen okra | ND | 0.05 ± 08 | ND | 0.03 ± 08 |
| Strawberry jam | 0.53 ± 08 | 0.61 ± 08 | 0.48 ± 06 | 0.58 ± 06 |

ND: not detected.

**TABLE 2**
**Atrazine Recovery and RSD Percentages of Spiked Food Samples Extracted by SFE and MSE, Determined by SFC at 220 nm and ELISA Technique at 450 nm**

| Food Samples | SFE | | MSE | |
|---|---|---|---|---|
| | SFC | ELISA | SFC | ELISA |
| Guava juice | 93.3 ± 1.2 | 98.7 ± 0.8 | 92.4 ± 1.6 | 97.4 ± 1.6 |
| Mango juice | 94.4 ± 1.1 | 97.2 ± 0.6 | 93.2 ± 1.3 | 97.1 ± 1.3 |
| Fruit cocktail juice | 94.2 ± 1.5 | 96.8 ± 0.7 | 94.8 ± 2.7 | 95.5 ± 2.7 |
| Frozen mixed vegetable | 93.8 ± 2.7 | 98.4 ± 1.2 | 93.4 ± 2.6 | 97.4 ± 1.6 |
| Frozen molukhia | 92.3 ± 1.6 | 97.2 ± 1.3 | 91.2 ± 2.3 | 96.2 ± 1.3 |
| Frozen artichoke | 95.4 ± 1.9 | 99.8 ± 1.7 | 93.8 ± 3.7 | 99.1 ± 2.4 |
| Frozen colocassia | 95.2 ± 1.3 | 97.4 ± 1.6 | 95.0 ± 3.6 | 96.7 ± 1.6 |
| Frozen beans | 92.8 ± 2.7 | 98.2 ± 1.5 | 92.2 ± 2.3 | 97.3 ± 1.3 |
| Frozen aubergines | 95.4 ± 1.6 | 99.8 ± 1.3 | 94.8 ± 2.6 | 98.8 ± 2.7 |
| Frozen okra | 92.2 ± 1.3 | 95.4 ± 1.2 | 91.4 ± 1.9 | 95.2 ± 2.5 |
| Strawberry jam | 90.8 ± 2.7 | 92.9 ± 1.8 | 89.1 ± 3.4 | 91.3 ± 2.6 |

**TABLE 3**
**MDL of Atrazine by SFC/UVD at 220 nm and ELISA Technique at 450 nm**

| Atrazine Concentration (ppb) | SFC | ELISA |
|---|---|---|
| 0.001 | ND | D |
| 0.03 | D | D |
| 0.06 | D | D |
| 0.09 | D | D |
| 0.1 | D | D |
| 0.5 | D | D |
| 1.0 | D | D |

ND: not detected, D: detected.

**TABLE 4**
**Overall Extraction and Determination Time by SFE, MSE, SFC, and ELISA Techniques of Each Food Sample**

| Extraction Time/Sample (min) | | Determination Time/Sample (min) | |
|---|---|---|---|
| SFE | MSE | SFC | ELISA |
| 45.0 | 2.7 | 5 | 1 |

Our results are consistent with other findings of 17 laboratories from 7 countries, which demonstrated that these methods meet the purpose of many monitoring programs for pesticide residue analysis in foods. For example, by using[18] a direct sample introduction (DSI), or "dirty sample injection", in the determination of 22 diverse pesticide residues in mixed apple, green bean, and carrot extracts by benchtop gas chromatography/tandem mass spectrometry (DSI/GC/MS-MS), similar results were obtained[18]. However, in this particular test, besides the targeted pesticides, some other chemicals were incurred in the samples including: chlorpyrifos, azinphos-methyl, parathion-methyl, diazinon, terbufos, p,p'-DDE, endosulfan sulfate, carbofuran, carbaryl, propargite, bifenthrin, dacthal, trifluralin, metalaxyl, pendimethalin, atrazine, piperonyl butoxide, diphenylamine, vinclozolin, chlorothalonil, quintozene, and tetrahydrophthalimide (the breakdown product of captan)[18]. The analytical DSI method entailed the following steps:

1. Blend 30-g sample with 60 ml acetonitrile for 1 min in a centrifuge bottle
2. Add 6 g NaCl and blend 30 sec
3. Centrifuge for 1–2 min
4. Add 5 ml upper layer to 1 g anhydrous $MgSO_4$ in a vial
5. Analyze 11 µl extract, using DSI/GC/MS-MS

In this method, sample cleanup is not needed because GCIMS-MS is exceptionally selective for the targeted analytes, and nonvolatile coextracted matrix components do not contaminate the injector or the GC/MS-MS system. Average recoveries of the pesticides were 103 ± 7% with RSDs of 14 ± 5% on average, and limits of detection were <2 ng/g for nearly all pesticides studied. The DSI/GC/ MS-MS approach for targeted pesticides is quantitative, confirmatory, sensitive, selective, rugged, rapid, simple, and inexpensive. Most of the current herbicide residue extraction methods are time consuming and solvent intensive. SFE using $CO_2$ has been used as an alternative for other residue extraction methods as a replacement for hazardous organic solvents. Atrazine recovery averaged 90.4% with an RSD of 3.3%. Hence, SFE is a viable technique for isolating atrazine residues from most foods and vegetables, requiring only 8 ml of solvent for each analysis[18,19,20,21,22,23].

## CONCLUSIONS

The atrazine residues studied are used frequently in agriculture as herbicides. The two extraction methods by SFE and MSE coupled with ELISA techniques were fast, safe, inexpensive, and had a high recovery percentage for all four groups of pesticide residues. With the SFE multiresidue method, the optimum conditions were met to extract 35 pesticides in each freeze-dried food sample (control or spiked) in 55 min as described previously in SFE method. ELISA determination method was fast, no cleanup required, and it has a low detection limit (0.001 ppb) when compared with SFC/UVD.

## BIOSKETCHES

**Mohamed El-Saeid, Ph.D.,** is a Visiting Professor in the Department of Chemistry, Texas Southern University, Houston. Mohamed El-Saeid was Assistant Professor Food Chemistry in the Department of Food Science and Technology, Faculty of Agriculture at Al-Azhar University in Egypt. E-mail: elsaeid03@yahoo.com

**Ijeoma Kanu, M.Sc., Ph.D.** (Candidate), is a lecturer in the Department of Microbiology, Abia State University and a Ph.D. Research Student in environmental microbiology at the Michael Okpara University of Agriculture, Umudike, Umuahia, Abia State, Nigeria. E-mail: ijay2001kay@yahoo.com

**Ebere C. Anyanwu, Ph.D.,** is a Clinical Neurophysiologist and a full-time Research Scientist in the Medical Center for Immune and Toxic Disorders, Spring, Texas. He was formally the Director of Cahers Neurosciences Research Inc, Houston and an Adjunct Professor of Anatomy and Physiology at the North Harris and Montgomery Colleges in Houston and The Woodlands, respectively. Dr. Anyanwu is also a Researcher at the Biomarkers Research Laboratory, Department of Environmental Chemistry and Toxicology, Texas Southern University, Houston. E-mail: ebereanyanwu@msn.com

**Mahmoud A. Saleh, Ph.D.** is a Professor of Chemistry and the Director of the Biomonitor Research Group, Department of Chemistry, Texas Southern University, Houston. Professor Saleh was the Chairman of the Department of Chemistry and has supervised several Ph.D. students.
